# Innovative thin endoscopic combination surgery in upper gastrointestinal tract: A case of endoscopic submucosal dissection and endoscopic hand suturing for gastric tumor

**DOI:** 10.1055/a-2408-8747

**Published:** 2024-10-02

**Authors:** Takuma Okamura, Tomonari Ikeda, Tatsuki Ichikawa, Hisamitsu Miyaaki

**Affiliations:** 1 13650 Department of Gastroenterology, Nagasaki Harbor Medical Center, Nagasaki, Japan; 2 200674 Department of Comprehensive Community Care Systems, Nagasaki University Graduate School of Biomedical Sciences, Nagasaki, Japan; 3 200674 Department of Gastroenterology and Hepatology, Nagasaki University Graduate School of Biomedical Sciences, Nagasaki, Japan

We previously reported the usefulness of a novel innovative endoscopic technique, thin endoscopic combination surgery, for rectal tumors 1 . Unlike in the lower gastrointestinal tract, double-scope procedures in the upper gastrointestinal tract have only been reported to date using a combination of a standard-diameter therapeutic endoscope and an ultrathin-diameter endoscope, owing to luminal restrictions in the pharynx and esophagus 2 3 . However, this combination allows for limited manipulation owing to the weak stiffness of the ultrathin-diameter endoscope and the small forceps channel. In contrast, thin endoscopic combination surgery allows for the insertion of two therapeutic endoscopes of the same thin diameter and a forceps channel measuring 3.2 mm, minimizing interference between scopes even in the upper digestive tract and allowing independent and coordinated use of all currently available instruments, as in laparoscopic surgery. In this report, we describe the endoscopic submucosal dissection (ESD) and endoscopic hand suturing (EHS) of gastric tumors using thin endoscopic combination surgery ( Video 1 ).

The innovative thin endoscopic combination surgery for endoscopic procedures.Video 1

The patient was an 84-year-old woman who was referred to our hospital for the treatment of a 0-IIa lesion measuring 20 mm in size in the middle to upper gastric body ( Fig. 1 **a–b** ). Thin endoscopic combination surgery was performed as previously described ( Fig. 2 **a–d** ). The resection was safely performed in 80 minutes ( Fig. 1 **c** ). Mucosal defect closure after resection was performed using EHS ( Fig. 1 **d** ). The operator was responsible for manipulating the needle using the SutuArt (Olympus, Tokyo, Japan), and the assistant was responsible for pulling up the tissue at the suture site with biopsy forceps to facilitate suturing or receiving the inserted needle, which can be easily adjusted without placing it on the mucosa by loosening the SutuArt and turning the thread, as is done in laparoscopic surgery ( Fig. 3 **a–d** ). Complete closure of the mucosal defect was achieved.

In conclusion, thin endoscopic combination surgery has the potential to improve the accuracy and simplicity of procedures for resection and defect closure.

**Fig. 1:**
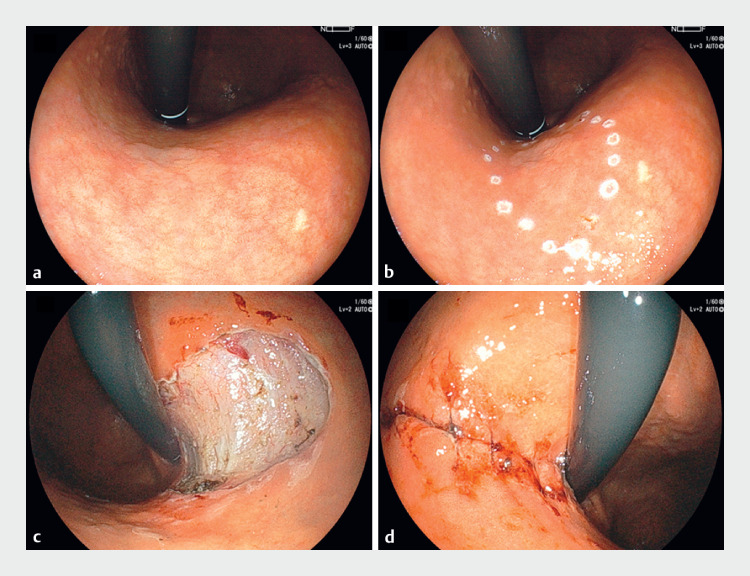
Endoscopic view of the findings before and after endoscopic submucosal dissection. **a** White light image of the gastric tumor. **b** Markings. **c** Post-endoscopic resection. The resected area measured 112 × 104 mm. **d** Complete closure was achieved using endoscopic hand suturing.

**Fig. 2:**
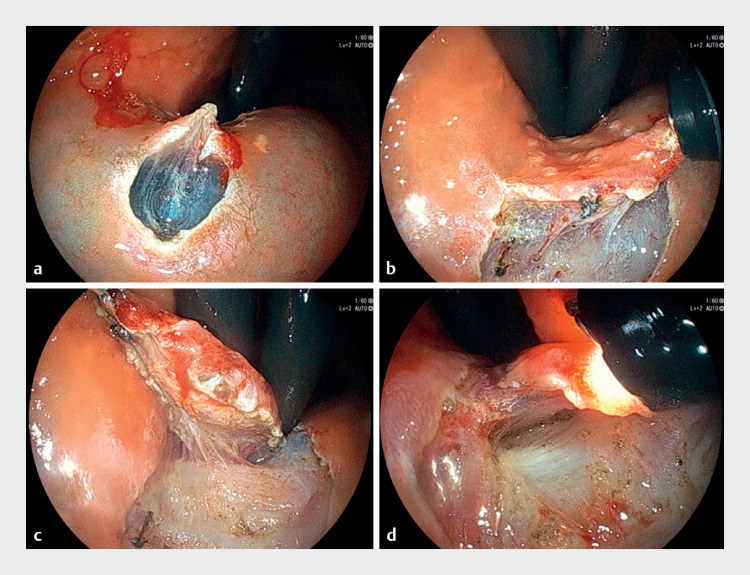
Endoscopic view of submucosal dissection with thin endoscopic combination surgery. **a–d** The assistant can grasp and apply traction to the tissue with biopsy forceps in the right direction at the right time, allowing the surgeon to perform safe and rapid resection.

**Fig. 3:**
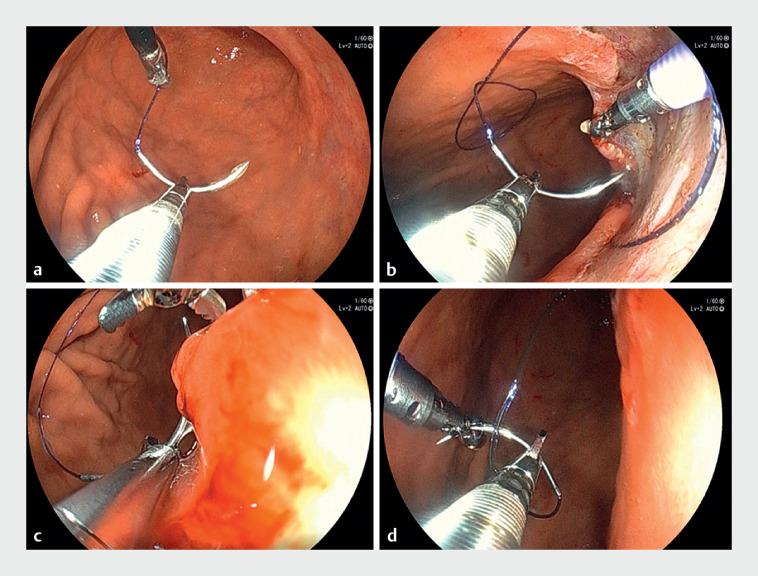
Endoscopic view of endoscopic hand suturing with thin endoscopic combination surgery. **a** The operator grasps the needle gently and adjusts the angle of the needle while an assistant rotates the thread. **b** The assistant pulls up the next suture site, making it easier for the surgeon to suture and reducing the risk of injury to extracanalicular organs. **c** After the needle is inserted with a single endoscope, the needle is often released and lost while retrieving it; however, with thin endoscopic combination surgery, an assistant can receive the needle and suture it quickly. **d** After the needle is removed, the assistant hands over the needle to the operator, eliminating the need to pick the needle up.

Endoscopy_UCTN_Code_TTT_1AO_2AG_3AD

## Notes

Endoscopy E-Videos https://eref.thieme.de/e-videos
*E-Videos* is an open access online section of the journal *Endoscopy* , reporting on interesting cases and new techniques in gastroenterological endoscopy. All papers include a high-quality video and are published with a Creative Commons CC-BY license. Endoscopy E-Videos qualify for HINARI discounts and waivers and eligibility is automatically checked during the submission process. We grant 100% waivers to articles whose corresponding authors are based in Group A countries and 50% waivers to those who are based in Group B countries as classified by Research4Life (see: https://www.research4life.org/access/eligibility/ ). This section has its own submission website at https://mc.manuscriptcentral.com/e-videos .
